# Wrist deformity, bother and function following wrist fracture in the elderly

**DOI:** 10.1186/s13104-020-05013-5

**Published:** 2020-03-20

**Authors:** Andrew Lawson, Partiban Santhakumar, Justine M. Naylor, Tim Churches, Steve Frost, Ian A. Harris

**Affiliations:** 1 Whitlam Orthopaedic Research Centre, Ingham Institute for Applied Medical Research, South Western Sydney Clinical School, UNSW, Sydney, NSW Australia; 2 Ingham Institute for Applied Medical Research, South Western Sydney Clinical School, UNSW, Sydney, NSW Australia; 3 Centre for Applied Nursing Research, Ingham Institute of Applied Medical Research, Sydney, NSW Australia; 4 South Western Sydney Local Health District (SWSLHD), Sydney, NSW Australia

**Keywords:** Bother, Wrist, Conservative treatment, Deformity, Fracture fixation, Patient reported outcome, Distal radius fracture

## Abstract

**Objective:**

Wrist deformity in older people is common following treatment for a wrist fracture, particularly after non-surgical treatment. A cohort of older wrist fracture patients were surveyed by telephone regarding perceived deformity, bother with deformity and patient-reported wrist function. The objectives were to: (1) determine whether older patients with wrist fractures perceived a deformity of their wrist and if they were bothered by it; (2) test if there were associations between deformity and treatment-type and between deformity and function; (3) test for associations between bother and treatment-type and between bother and function; (4) measure the test–retest reliability of the ‘bother’ question.

**Results:**

Of 98 eligible patients who were invited to participate, 41 responded. Out of 41, 14 (34%) believed they had a deformity and 4 (10%) reported that they were bothered by the appearance of their wrist. Deformity was associated with non-surgical treatment (RR = 3.85, p = 0.006) but was not significantly associated with functional outcomes (p = 0.15). All those who were bothered belonged to the non-surgical treatment group. Bother was significantly associated with poorer functional outcomes (p = 0.006) and this association was clinically significant (MD = 35 points). The deformity and bother questions were found to have excellent test–retest reliability; κ = 1.00 and κ = 0.92, respectively.

## Introduction

Wrist fractures are common in people aged 50 and over. Wrist fractures are associated with female gender, simple falls and osteoporosis [1–3]. Wrist fractures account for 18% of all fracture types in the elderly (65 years and over) [4]. The two most common forms of treatment for wrist fractures in older patients are non-surgical treatment (closed reduction and plaster immobilization) and surgical fixation using a volar locking plate [5, 6]. Traditionally, in older patients, wrist fractures were most commonly managed by non-surgical treatment. However, there has been a shift towards surgical treatment (particularly volar locking plate fixation) with a fivefold increase in the past 20 years [5]. The main reason for the use of surgical fixation is to maintain better fracture alignment. A survey of orthopaedic surgeons in Australia who were presented with a case of a typical wrist fracture sustained by a 75-year-old female revealed stronger preference for surgical fixation using volar locking plates (47%) compared with closed reduction with cast immobilisation (23%) [7].

Malunion is a common complication following non-surgical treatment of wrist fractures. Estimates of rates of malunion associated with closed reduction and cast fixation of wrist fractures have been reported as high as 50% [1, 2]. Symptomatic deformity can be treated using surgery [8]. However, malunion and the presence of deformity are usually well-tolerated and deformity isn’t necessarily associated with poor functional outcomes. In fact, studies comparing functional outcomes with radiographic outcomes in the medium and long-term following treatment for wrist fracture have shown little if any agreement [9–11]. Despite the higher rates of malunion and deformity associated with closed reduction, trials comparing surgical and non-surgical treatment of wrist fractures in elderly patient populations have demonstrated that satisfactory functional outcomes can still be achieved with non-surgical treatment [1, 12, 13].

Understanding the degree to which patients experience and are bothered by wrist deformity following the treatment of their fractured wrist would assist in clinical decision making for this common fracture. The aims of this retrospective study were fourfold. We aimed to investigate:

1. Incidence of perceived deformity and bother and their association.
2. Association between perceived deformity, and treatment and function.
3. Association between bother, and treatment and function.
4. Test–retest reliability of deformity and bother questions.

## Main text

### Methods

#### Patient population and screening

The patient population were elderly people who had presented to our institution over a 5-year period with a wrist fracture. Eligible people were identified from an electronic orthopaedic database. Inclusion criteria were: age 65 years and over; treatment of their fracture either by closed reduction and cast immobilization or by open reduction and plate fixation. Exclusion criteria included residency in a nursing home and inability to provide informed consent (e.g. those patients with documented dementia or those with low English proficiency).

Potentially eligible participants were sent a letter informing them of the study (Additional file 1: Appendix S1) and providing them with the opportunity to opt out by contacting the research department. After 2–3 weeks, a follow-up telephone call was made to eligible participants who had not opted out. Efforts were made to contact the entire cohort. If a telephone number could not be found, then a second letter was sent to the eligible participant inviting them to contact the research institute if they were interested in participating in the study.

Those agreeing to participate provided consent and the survey was conducted over the telephone by a researcher (PS).

#### Outcome measures

The telephone survey (Additional file 2: Appendix S2) was made up of two parts. The first part of the survey was designed to assess patient-perceived deformity and bother. Firstly, participants were asked whether they believed that they had a deformity in the affected wrist using a simple dichotomous question; “Do you consider your wrist to be deformed or crooked?” Secondly, participants were asked about the extent to which they were bothered by the appearance of their wrist on a 5-point Likert scale (1—Not at all, 2—A little, 3—Moderately, 4—Very, 5—Extremely). The format of the bother question was based on the format of questions that make up the bother index in the short musculoskeletal function assessment (SMFA) questionnaire. The SMFA has been assessed as being a reliable and valid tool for use in musculoskeletal disease or injury [14].

The second part of the survey consisted of the Patient-Rated Wrist Evaluation (PRWE) questionnaire. The PRWE is a common and validated measure that is used to assess the pain and function of a patient following a wrist injury [15]. The PRWE is made up of two sections assessing pain and function. The sum of both the pain and function sections produces a single score out of 100 [16], with 0 representing no wrist pain and disability and 100 representing maximum wrist pain and disability. The minimum clinically important difference (MCID) for PRWE scores for wrist fracture patients has been reported as 11.5 points [17].

To assess the reliability of the deformity and the bother questions, participants were asked for their consent to be followed up 1 week after the initial phone interview to be asked the two questions again. The aim of this was to assess the test–retest reliability of these two questions. See Additional file 1: Appendix S1 for entire telephone script including the survey questions.

#### Statistical analysis

Results were analysed using R-statistical language [18].

Relative risk ratios (RR) and p-values were reported for perceived deformity by treatment-type and for perceived deformity vs bother.

As an indication of wrist function, PRWE scores were calculated. Medians and interquartile ranges of PRWE results were reported, according to associations with treatment-type, perceived deformity and perceived bother. Statistical significance was measured using chi squared tests and Mann–Whitney Wilcoxon tests (*U*). Means of functional scores were compared between groups using one-way analysis of Variance (ANOVA). Results were statistically significant when p < 0.05.

The test–retest reliability of the deformity and bother questions was analysed using Cohen’s Kappa. Kappa scores between 0.75 and 1.00 were considered to be excellent [19].

### Results

#### Cohort ascertainment

**Fig. 1:**
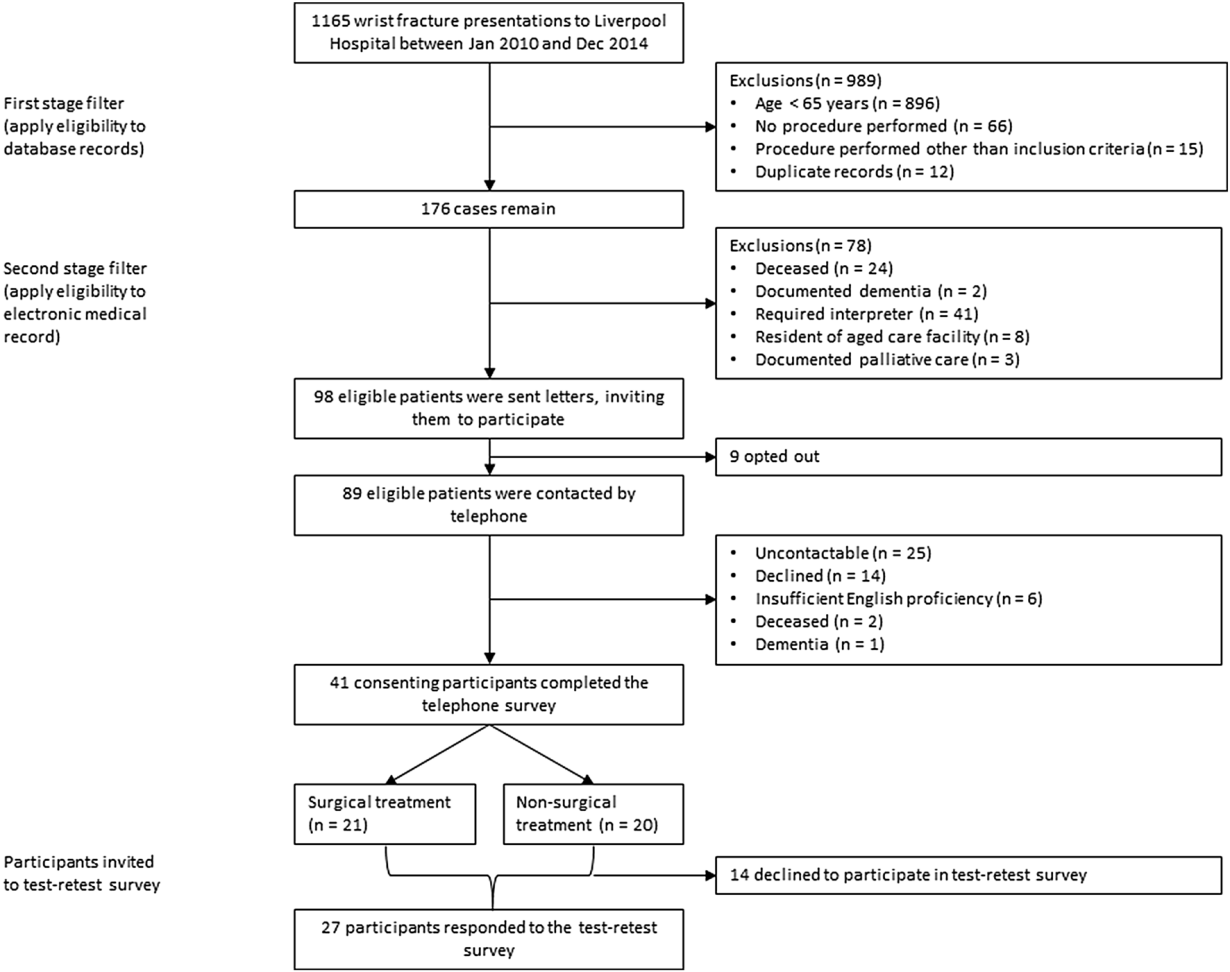
Flowchart of inclusion of participating patients

From 1165 recorded cases of wrist fracture presenting between January 2010 and December 2014, there were 98 potentially eligible participants. 9 opted out and 89 eligible patients were contacted by telephone. From these, 41 consented to undertake the initial survey and 27 of these consented to repeating the survey 1-week later. A profile of the study participants is described in Additional file 3 and the participant flow is displayed in Fig. 1. Participant characteristics were similar to the cohort with regard to age, treatment-type and injured side (left or right). There was a low participation rate of males compared with the cohort.

#### Incidence of perceived deformity and bother

Of 41 participants, 14 (34%) perceived that they had a wrist deformity and 4 (10%) reported that they were bothered by the appearance of their wrist. Three of these four perceived that they had a wrist deformity. Interestingly, one participant who did not perceive a deformity reported that he or she was bothered by the appearance of his or her wrist. Additional file 4 displays a comparison of the perception of bother (dichotomous; yes/no) with the perception of deformity (yes/no). Those who perceived that they had a deformed wrist were more likely to be bothered by the appearance of it than those who perceived no deformity (RR 5.79, p = 0.07).

#### Association between perceived deformity and treatment-type/function

In the non-surgical treatment group, 11 out of 20 patients (55%) perceived deformity and in the surgical treatment group, only 3 out of 21 patients (14%) believed that their wrist was deformed. Participants treated non-surgically were more likely to perceive deformity compared to those treated surgically (RR 3.85, p = 0.006).

**Fig. 2:**
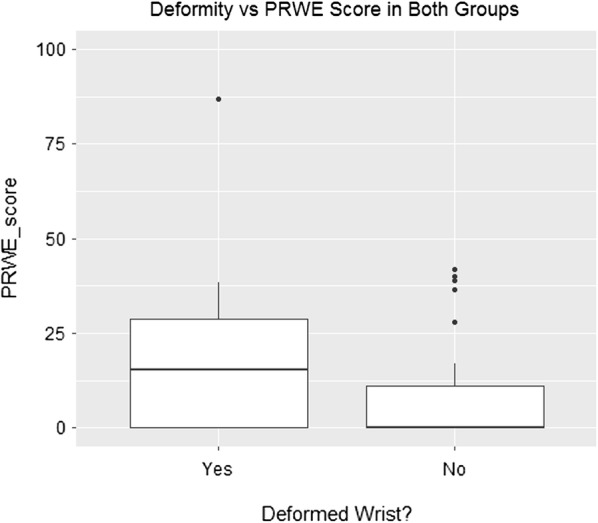
Distribution of functional scores by perceived deformity

Figure 2 and Additional file 5 display the distribution of PRWE scores according to whether deformity was perceived. Overall, functional outcomes were poorer for those who perceived deformity, but this was not statistically significant (U = 239, p = 0.15). The mean difference (MD) in PRWE scores between those who perceived deformity and those who did not perceive deformity was 10.9 which was close to the MCID of 11.5 points.

#### Association between bother and treatment-type/function

All participants who reported that they were bothered by wrist deformity belonged to the non-surgical treatment-type group.

**Fig. 3:**
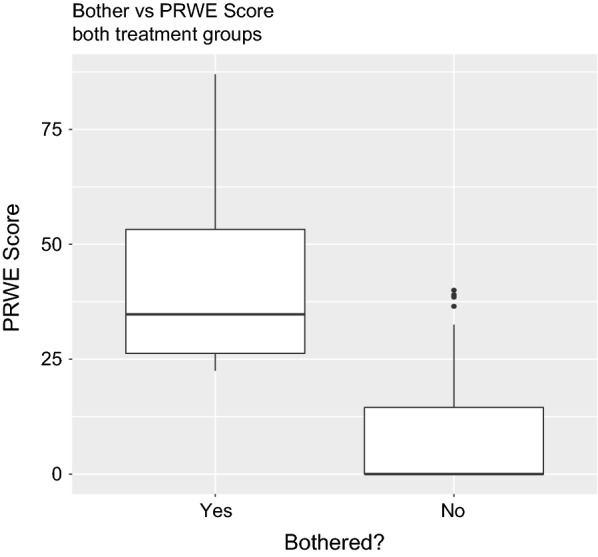
Distribution of functional scores by reported bother (yes/no)

Figure 3 and Additional file 6 display the distribution of PRWE scores by whether bother was reported. There were only four participants who reported that they were bothered by the appearance of their wrist and their functional outcomes were poorer than those who reported that they were not bothered, by both a statistically significant (U = 14, p = 0.006) and by a clinically important (MD = 35.2) margin.

Additional file 7 displays the degrees of bother. Additional file 8 is a scatterplot displaying the distribution of functional scores according to the extent of bother reported. The correlation between the extent of bother and functional scores was significant (r = 0.44, p = 0.004).

#### Reliability of deformity and bother questions

For the deformity question, all 27 patients who participated in the test–retest component provided a consistent answer on retest, indicating perfect reliability (κ = 1.00) (Additional file 9). For the bother question, 26 participants provided the same answer week-to-week and one changed their answer from a little bothered to not at all bothered. The reliability of the bother question at κ = 0.92 represented “excellent agreement”.

### Discussion

The existing evidence concerning wrist deformity following treatment of wrist fractures suggests that deformity is common and that patients are not troubled or bothered by the deformity: A 2005 review of the predictors of patient satisfaction following treatment for wrist fracture concluded that satisfaction was associated with pain relief and grip strength rather than the radiographic findings [20]. A 2011 randomised controlled trial [12] comparing surgical and non-surgical treatment of wrist fractures collected deformity and bother as outcomes but the results were not presented in the report. A 2000 retrospective study on wrist fractures in older patients reported that despite obvious clinical deformity in half of their cohort (14/25), none were “dissatisfied” with the appearance of their wrist [21].

The study most comparable to the current study was a 2009 study of patients over 70 years of age who had undergone surgical or non-surgical treatment for an unstable wrist fracture. That study found that none of the surgical patients had a deformity and that 77% of non-surgical patients had a visual deformity [22]. However, the authors reported that no participant was “dissatisfied with the clinical appearance” of their wrist. In comparison, we found a lower rate of deformity (14/41) and we found that some patients with perceived deformity were bothered by it (4/41). The difference is likely explained in that our study used patient-reported deformity rather than therapist-assessed deformity.

## Limitations

This study had a few limitations. Primarily, the sample was small potentially limiting the ability to detect statistically significant small differences, and the response rate was low limiting the generalizability of our findings. The study used a convenience sample which was small because the inclusion criteria (treatment-type and age range) were narrow. There were 98 eligible patients but our response rate yielded a sample of only 41. To be able to observe the effect that we observed in function by perceived deformity to a level of significance, we would need a sample size of over 40 in each treatment group. Also, the bother question was designed as a Likert scale but given that only four participants reported that they were bothered by the appearance of their wrist, the Likert format added little information to the dichotomous format. In a larger cohort, the Likert format may have proved meaningful in informing the extent of bother.

Further, the study was retrospective. As such, the treatment-type was not controlled and the time to follow-up ranged between 2 and 5 years. This created a potential for indication bias relating to the comparisons made between treatment groups.

Finally, the mode of administration may be a limitation. Telephone administration of the survey by an investigator helped ensure the completeness and accuracy of data collected. However, it also created the potential for observer bias. This potential was minimized by using a telephone script (see Additional file 1: Appendix S1).

## Supplementary information

**Additional file 1: Appendix S1.** Patient information sheet.

**Additional file 2: Appendix S2.** Phone script.

**Additional file 3.** Participant profile.

**Additional file 4.** Deformity vs bother.

**Additional file 5.** Distribution of functional scores by perceived deformity.

**Additional file 6.** Distribution of functional scores by reported bother (yes/no).

**Additional file 7.** Deformity vs Degree of Bother.

**Additional file 8.** Scatter plot for degree of bother and function scores.

**Additional file 9.** Reliability of bother question.

## Data Availability

The datasets used and/or analysed during the current study are available from the corresponding author on reasonable request.

## Abbreviations

ANOVA: Analysis of variance
MCID: Minimal clinically important difference
MD: Mean difference
PRWE: Patient-rated wrist evaluation
RR: Risk ratio
SMFA: Short musculoskeletal function assessment
