# Supplementary material for: Wrist deformity, bother and function following wrist fracture in the elderly
Source: BMC Res Notes. 2020 Mar 20;13:169. doi: 10.1186/s13104-020-05013-5 (PMC7085157; doi:10.1186/s13104-020-05013-5)
Supplement: Supplementary file 1 — Additional file 1: Appendix S1. Patient information sheet. [file 13104_2020_5013_MOESM1_ESM.docx]

(Letterhead)

Date: (Insert date)

To: (Insert patient name)

(Insert address line 1)

(Insert address line2)

Dear (Insert patient name),

Re: **Research on people with wrist fractures**.

We are part of a research team from the University of NSW based at Liverpool Hospital, and are conducting research on how much patients are bothered by their wrist after having treatment for a wrist fracture. You have been selected as a potential participant in this research as you had a wrist fracture treated in plaster at Liverpool Hospital in the last few years.

You are invited to participate in this research which will involve a telephone survey which will be completed by one of our research students. This will be a 5 minute survey which will ask about any issues you’ve had following your wrist injury as well as the current level of pain and function. We will contact you by telephone in the next few weeks to conduct the survey.

If you agree to participate, information from your hospital records relating to your wrist fracture treatment will also be used for the purposes of this research. All information collected will be kept strictly confidential, and will only be used for research purposes. Your participation in this research will not affect your relationship with the health service. You may also be contacted at a future date to be asked about your wrist. We cannot and do not guarantee that you will receive any benefits from this study. Participation is voluntary and you are free to withdraw at any time. If you do not participate, this decision will not affect your current or future treatment.

If you do not wish to be called regarding this research or if you have any enquiries please contact the Orthopaedic Department secretary on (02) 9828 3898. The study has been approved by our local ethics committee. If you have concerns about the research, you can contact the Ethics Committee at Liverpool Hospital on XXXX XXXX or on email at XXXXXX.

Regards,

Professor Ian Harris

Professor of Orthopaedic Surgery, University of New South Wales

Director of Orthopaedics, Liverpool Hospital
