# Supplementary material for: Wrist deformity, bother and function following wrist fracture in the elderly
Source: BMC Res Notes. 2020 Mar 20;13:169. doi: 10.1186/s13104-020-05013-5 (PMC7085157; doi:10.1186/s13104-020-05013-5)
Supplement: Supplementary file 2 — Additional file 2: Appendix S2. Phone script. [file 13104_2020_5013_MOESM2_ESM.docx]

**Appendix S2**

*Patient problems with deformity after distal radius fractures – telephone interview transcript.*

*Version 2, 5 December 2015*

Hello, may I speak with [PATIENT NAME]?

*If not available, ask when they would be available to take a call, of would they prefer to call back*

*Once connected:*

My name is _________ from the University of New South Wales and I am currently working with Professor Harris at a research centre in Liverpool. We are currently doing a survey of patients who have had wrist fractures treated at Liverpool Hospital. You may have received a letter about this research in the mail.

*Pause for any response*

We are interested in whether or not people have any problems with the appearance of their wrist after having a fracture treated. You have been contacted as you had a fracture of the wrist treated at Liverpool Hospital in the past. We would like to ask you a few questions about your wrist – in particular, about whether the wrist is giving you any problems. There are just a few questions and it will only take a few minutes to answer them. Are you happy for me to ask you those questions now?

*Pause for response. If ‘no’, thank them for their time. If ‘yes’:*

OK, thanks. The first question is:

1- Do you consider your wrist to be deformed or crooked?

(Yes, no, unsure?)

2- On a scale from 1 to 5, how much are you bothered by the appearance of your wrist? (1 being not at all bothered and 5 being extremely bothered)

(1-Not at All, 2-A Little, 3-Moderately, 4-Very, 5-Extremely)

Now I am going to ask you some questions that relates to your current wrist pain and function.

Q3-17 – Ask 15 questions from the PRWE

Part of the purpose of our study is to test the repeatability of the questions about whether you are bothered by your wrist. Would you be happy if we contacted you a week from now to ask you the first 2 questions again? It will take about 2 minutes.

(If ‘ok’, make a time to call next week)

That’s all for today and thank you for your time.
