# Supplementary material for: Wrist deformity, bother and function following wrist fracture in the elderly
Source: BMC Res Notes. 2020 Mar 20;13:169. doi: 10.1186/s13104-020-05013-5 (PMC7085157; doi:10.1186/s13104-020-05013-5)
Supplement: Supplementary file 3 — Additional file 3. Participant profile. [file 13104_2020_5013_MOESM3_ESM.docx]

**Additional file 3**

**Wrist deformity, bother and function following wrist fracture in the elderly**

**Additional file 3; participant profile (n=41)**

|  |  | Participants (n=41) | All patients contacted (n=89) |
| --- | --- | --- | --- |
| Age (years) | Median, (IQR) | 71 (68, 76) | 71 (68, 77.5) |
| Sex | Female; n (%) | 39 (95.1) | 75 (84.3) |
|  | Male; n (%) | 2 (4.9) | 14 (15.7) |
| Method of treatment | Closed reduction; n (%) | 20 (48.8) | 44 (49.4) |
|  | Open reduction; n (%) | 21 (51.2) | 45 (50.6) |
| Injured wrist | Left; n (%) | 24 (58.5) | 48 (53.9) |
|  | Right; n (%) | 17 (41.5) | 40 (45.0) |
|  | Unknown; n (%) | 0 (0) | 1 (1.1) |
| Time since injury |  | 2 – 5 years | 2 – 5 years |
