# Supplementary material for: Wrist deformity, bother and function following wrist fracture in the elderly
Source: BMC Res Notes. 2020 Mar 20;13:169. doi: 10.1186/s13104-020-05013-5 (PMC7085157; doi:10.1186/s13104-020-05013-5)
Supplement: Supplementary file 4 — Additional file 4. Deformity vs bother. [file 13104_2020_5013_MOESM4_ESM.docx]

**Additional file 4**

**Wrist deformity, bother and function following wrist fracture in the elderly**

**Additional file 4: deformity vs bother**

| Perceived Deformity | Bother | | Total | Incidence |
| --- | --- | --- | --- | --- |
|  | Yes | No |  |  |
| Yes | 3 | 11 | 14 | 3/14 = 0.21 |
| No | 1 | 26 | 27 | 1/27 = 0.04 |
| Relative risk = 5.79 (95%CI, 0.66, 50.63), p = 0.07 | | | | |
