# Supplementary material for: Wrist deformity, bother and function following wrist fracture in the elderly
Source: BMC Res Notes. 2020 Mar 20;13:169. doi: 10.1186/s13104-020-05013-5 (PMC7085157; doi:10.1186/s13104-020-05013-5)
Supplement: Supplementary file 5 — Additional file 5. Distribution of functional scores by perceived deformity. [file 13104_2020_5013_MOESM5_ESM.docx]

**Additional file 5**

**Wrist deformity, bother and function following wrist fracture in the elderly**

**Additional file 5; distribution of functional scores by perceived deformity**

| Deformity | Yes | No |
| --- | --- | --- |
| N | 14 | 27 |
| Mean | 20.14 | 9.22 |
| SD | 23.55 | 14.39 |
| Min | 0 | 0 |
| P25 | 0 | 0 |
| P50 | 15.25 | 0 |
| P75 | 28.63 | 11 |
| Max | 87 | 42 |
| U = 239  P = 0.15 | | |
