# Supplementary material for: Wrist deformity, bother and function following wrist fracture in the elderly
Source: BMC Res Notes. 2020 Mar 20;13:169. doi: 10.1186/s13104-020-05013-5 (PMC7085157; doi:10.1186/s13104-020-05013-5)
Supplement: Supplementary file 6 — Additional file 6. Distribution of functional scores by reported bother (yes/no). [file 13104_2020_5013_MOESM6_ESM.docx]

**Additional file 6**

**Wrist deformity, bother and function following wrist fracture in the elderly**

**Additional file 6; distribution of functional scores by reported bother (yes/no)**

| Bother | Yes | No |
| --- | --- | --- |
| N | 4 | 37 |
| Mean | 44.75 | 9.51 |
| SD | 29.36 | 13.57 |
| Min | 22.50 | 0 |
| P25 | 26.25 | 0 |
| P50 | 34.75 | 0 |
| P75 | 53.25 | 14.50 |
| Max | 87 | 40 |
| U = 14  P = 0.006 | | |
