# Supplementary material for: Wrist deformity, bother and function following wrist fracture in the elderly
Source: BMC Res Notes. 2020 Mar 20;13:169. doi: 10.1186/s13104-020-05013-5 (PMC7085157; doi:10.1186/s13104-020-05013-5)
Supplement: Supplementary file 7 — Additional file 7. Deformity vs Degree of Bother. [file 13104_2020_5013_MOESM7_ESM.docx]

**Additional file 7**

**Wrist deformity, bother and function following wrist fracture in the elderly**

**Additional file 7: Deformity vs Degree of Bother**

| Deformed Wrist | 1  (Not at all) | 2  (A little) | 3  (Moderately) | 4  (Very) | 5  (Extremely) |
| --- | --- | --- | --- | --- | --- |
| Yes | 11 | 1 | 1 | 1 | 0 |
| No | 26 | 1 | 0 | 0 | 0 |
| p = 0.031 | | | | | |
