# Supplementary material for: Wrist deformity, bother and function following wrist fracture in the elderly
Source: BMC Res Notes. 2020 Mar 20;13:169. doi: 10.1186/s13104-020-05013-5 (PMC7085157; doi:10.1186/s13104-020-05013-5)

**Additional file 8**

**Wrist deformity, bother and function following wrist fracture in the elderly**

**Additional file 8; scatter plot for degree of bother and function scores**
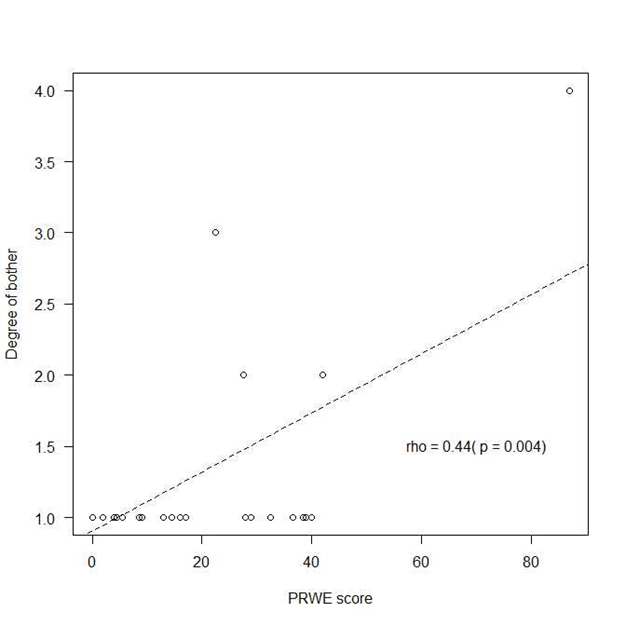

Supplement: Supplementary file 8 — Additional file 8. Scatter plot for degree of bother and function scores. [file 13104_2020_5013_MOESM8_ESM.docx]
