# Supplementary material for: Wrist deformity, bother and function following wrist fracture in the elderly
Source: BMC Res Notes. 2020 Mar 20;13:169. doi: 10.1186/s13104-020-05013-5 (PMC7085157; doi:10.1186/s13104-020-05013-5)
Supplement: Supplementary file 9 — Additional file 9. Reliability of bother question. [file 13104_2020_5013_MOESM9_ESM.docx]

**Additional file 9**

**Wrist deformity, bother and function following wrist fracture in the elderly**

| **Additional file 9: Reliability of bother question** | | Test 2 | |  |
| --- | --- | --- | --- | --- |
|  |  | Yes | No |  |
| Test 1 | Yes | 9 | 1 | 10 |
|  | No | 0 | 17 | 17 |
|  |  | 9 | 18 | 27 |
